# Link Functions in Multi-Locus Genetic Models: Implications for Testing, Prediction, and Interpretation

**DOI:** 10.1002/gepi.21635

**Published:** 2012-04-16

**Authors:** David Clayton

**Affiliations:** Juvenile Diabetes Research Foundation/Wellcome Trust Diabetes and Inflammation LaboratoryCambridgeInstitute for Medical Research, Cambridge UniversityUnited Kingdom

**Keywords:** epistasis, interaction, multi-locus models, prediction, tests for association

## Abstract

“Complex” diseases are, by definition, influenced by multiple causes, both genetic and environmental, and statistical work on the joint action of multiple risk factors has, for more than 40 years, been dominated by the generalized linear model (GLM). In genetics, models for dichotomous traits have traditionally been approached via the model of an underlying, normally distributed, liability. This corresponds to the GLM with binomial errors and a probit link function. Elsewhere in epidemiology, however, the logistic regression model, a GLM with logit link function, has been the tool of choice, largely because of its convenient properties in case-control studies. The choice of link function has usually been dictated by mathematical convenience, but it has some important implications in (a) the choice of association test statistic in the presence of existing strong risk factors, (b) the ability to predict disease from genotype given its heritability, and (c) the definition, and interpretation of epistasis (or epistacy). These issues are reviewed, and a new association test proposed. Genet. Epidemiol. 36:409–418, 2012. © 2012 Wiley Periodicals, Inc.

## INTRODUCTION

Multiple regression models, and the logistic regression model in particular, have a long history in the analysis of epidemiological studies of multifactorial disease. Since its introduction by Truett et al. (1967), this has become the preeminent statistical tool of the epidemiologist and only in the 1980s were other models explored in a systematic way, to examine the possibility of discriminating between different *mechanistic* models. A framework for such a formal study was provided by the idea of generalized linear models (GLMs) (McCullagh and Nelder, 1989; Nelder and Wedderburn, 1972)—regression models in which the scale on which effects of covariates combine in an additive manner can be varied by choice of a “link function.”

Just as epidemiologists in the late 1960s had to address the problem of multifactorial influences on disease, geneticists faced the same problems in addressing the genetics of “complex” traits in which disease is influenced by a multiplicity of genetic and environmental causes. In particular, Risch (1990) considered two different models for the manner in which multiple genes act together to influence risk of a complex disease trait and explored their implications in the context of linkage analysis. However, although the problems faced by geneticists and epidemiologists in studying multifactorial disease have much in common, there has been remarkably little reference to the earlier epidemiological literature in the more recent genetic literature (Clayton, 2009).

In this paper, these issues are reviewed in the context of modern genetic association studies, starting with the issue of whether it is necessary to allow for known risk factors when testing for new genetic associations. It is shown that the optimal test strategy depends on model which holds and that the concept of the “link function” of GLMs captures this dependence elegantly. Later sections deal with implications of the link function for interpretation in terms of mechanism, and for the potential to predict disease outcome from genotype.

## TESTING FOR ASSOCIATION IN THE PRESENCE OF KNOWN RISK FACTORS

This section deals with the problem of testing for association in the presence of known, strongly predictive, risk factors. For simplicity, it is assumed that existing factors can be represented by a discrete stratification, although a full regression generalization follows naturally. We start by revisiting the theory of the conventional stratified test within the general framework of the logistic regression model.

### THE LOGISTIC REGRESSION MODEL AND CASE-CONTROL STUDIES

We shall first introduce some notation. Let 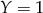 denote presence of disease and 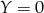 its absence, let *X* represent a single variable of interest and let *Z* represent covariates. Here, *Z* will simply represent a discrete variable describing risk strata, but the results presented may readily be generalized to the full regression case. If *S* represents the fact of being sampled in a case-control study and if *S* is independent of *X* and *Z*, Bayes theorem gives


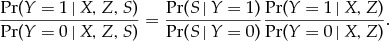


The first term on the right-hand side, which will be denoted by *K*, is the ratio of sampling rates of cases and non-cases or, equivalently


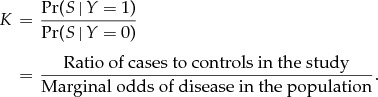


In general, *K* will be unknown, but it can usually be estimated approximately and, for the purpose required here, only an approximate estimate will be necessary. Taking logarithms,


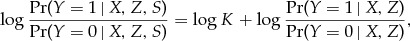


giving the well known result that, if a logistic regression model holds for disease in the population:


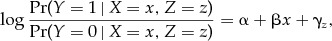


then a logistic regression model will also hold in the case-control study, with all coefficients unchanged save for the intercept, which becomes 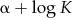. It is this result which has led to logistic regression having become the preeminent statistical tool of modern epidemiology.

The logistic regression model is a GLM with binomial (Bernoulli) error structure and logit link function and, since this link function is the “canonical” link in this setting, the first derivative of the log-likelihood with respect to the parameter β takes a very simple form. For observations 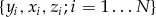, this is


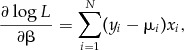


where 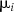 is the expectation of 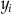 given by the model, given the values of *x*_1_ and 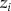. This expression gives the score test statistic for association between disease and exposure in the presence of stratification, proposed by Mantel (1963) as an extension to the Cochran-Armitage test for trend in proportions (Armitage, 1955; Cochran, 1954); one simply evaluates the above expression at the maximum-likelihood estimate of the remaining model parameters, having set 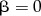. This simply replaces 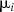 by the stratum means of *y*. More usually, the test is written in terms of the three-dimensional contingency table of frequencies, 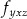,


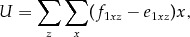


where a dot subscript denotes summation, and 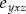 denote the “expected” frequencies under the null hypothesis of condition independence of *Y* and *X* given *Z*:


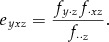


If we denote by 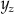 and 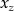 the vectors of observations in stratum *z* (where the correspondence of *y* and *x* observations of the same subject is maintained), the test may also be written as


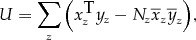


where 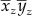 are the stratum specific means of *x* and *y*, respectively, and 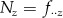, the number of subjects in stratum *z*. The variance formula given by Mantel is based on an argument which conditions on the marginal frequency tables 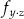 and 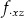. This is simply the permutation variance of *U* under random permutations of the order of the elements of 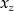 and 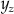. Thus,


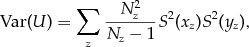


where *S*^2^() denotes the (biased) sample variance of its vector argument, 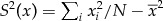. An “exact” test can be generated by the same permutation argument.

In the context of the case-control study, the logistic regression argument seems strange in that it conditions on *X* and *Z* and treats *Y* as a random outcome, while the study design suggests the reverse. However, Mantel's test conditions on *both* margins of the 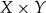 contingency table within each stratum, thus generalizing the argument of Fisher's exact test. That the asymptotic properties of the logistic regression approach to the analysis of case-control studies remain in force more generally, despite the reversal of conditioning was established by Prentice and Pyke (1979).

Exactly the same test can be generated from the standpoint of the log-linear model for contingency tables (Bishop et al., 1975; Goodman, 1970). The logistic regression model described above is implied by a log-linear model for the expected values of the frequencies, 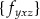, which includes all first-order associations:


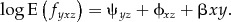


The parameter β in this model corresponds precisely with the parameter β of the logistic regression model above. The score test of H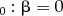 is as before, and its null distribution conditions on the sufficient statistics for ψ and ϕ, i.e., upon the marginal tables 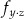 and 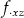. This corresponds to the same permutation argument as described above.

In genome-wide association studies, *X* represents a single nucleotide polymorphism (SNP) and has three levels. It is coded numerically, with the heterozygous genotype (Aa, say) coded at the midpoint between the values for the two homozygous genotypes (aa and AA). Thus, the alternative hypothesis holds that the odds ratio for Aa vs. aa is the same as that for AA vs. Aa, and is not equal to one. The test does not formally depend on this assumption, since it is evaluated under the null hypothesis 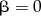, but this is the alternative hypothesis for which its power is maximized. However, as we shall see, the score test described above is also the score test for a wider class of alternatives in which the risk for the heterozygous genotype is intermediate between those for the homozygous genotypes, so that the test will be locally most powerful against this wider class. Perhaps reflecting this, this score test is widely used in the analysis of genome-wide association studies. Nevertheless, a two degree of freedom test against the wider class in which heterozygous risk is unrestricted may easily be derived by coding the SNP genotype as two indicator variables, *x*_1_ and *x*_2_ say. Such a test can be extended in exactly the same way as is proposed below for the one degree of freedom test.

### “A SURPRISING RESULT …”

A somewhat counter-intuitive aspect of this test was pointed out by Robinson and Jewell (1991), and has been discussed at some length in the epidemiological literature. Their “surprising result” is that, in a logistic regression analysis in which *X* and *Z* are conditionally independent given *Y* but *Y* and *Z* are associated, inclusion of *Z* in the regression results in a loss of power as compared with the an analysis which simply ignores *Z*. Robinson and Jewell contrasted this with the case of a classical regression model where *Y* is a continuous, normally distributed response and *X* and *Z* are *marginally* independent, where the reduction in residual variance achieved by including *Z* in the model results in an *increase* in power. This latter situation fosters a strong intuition that one should always adjust for a covariate which is strongly related to response, even if it is not related to the predictor variable of interest. As is now well appreciated in the epidemiological literature, Robinson and Jewell's result shows that this is not the case for the analysis of case-control studies, where the sampling scheme leads to *conditional* independence of *X* and *Z* given disease status, *Y*. Here, the use of the stratified test can result in appreciable loss of power.

This has been noted by Ling Kuo and Feingold (2010) in the context of genetic association studies, where it is particularly relevant. For example, in type 1 diabetes there is a long-established and very strong association with certain human leukocyte antigen (HLA) types yet, with the exception of loci close to the major histocompatibility complex (MHC) region on chromosome 6, other genetic loci would not be expected to be related to HLA genotype at a population level. An example of an environmental covariate would be cigarette smoking in genetic association studies of lung cancer; again, we would expect most loci to be unrelated to smoking behaviour. While the loss of power resulting from the use of stratified tests can be avoided by *matching* in the design of the case-control studies, many genome-wide association studies make use of readily available, general purpose control groups.

Some intuition concerning the loss of power due to stratification, and a clue to its possible recovery, are evident from consideration of the log-linear model. The log-linear model considered above which, when after conditioning on *X* and *Z* is equivalent to the logistic regression model, includes a term for association between *X* and *Z* (represented by the parameter set 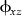). If, under the null hypothesis, we may assume independence of *X* and *Z*, then this term can be omitted from the model. Then, in the expression for the expected frequencies 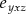, the ratios 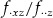, which estimate the distribution of *X* conditional on *Z*, can be replaced by the *marginal* estimates 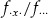. Then the test statistic simplifies to


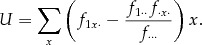


This is the usual Cochran-Armitage test statistic, and it ignores the stratification, *z*. It can also be written 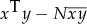, where omission of the *z* subscript indicates disregarding of the stratification, and the appropriate argument for generating the variance of the test statistic (or an exact *P*-value) is permutation of the elements of *x* and *y*. In the special case where *X* is dichotomous, the test becomes Fisher's exact test for association in the 2 × 2 table.

Thus, taking account of the independence of *X* and *Z* in the model avoids the loss of power of the stratified test as compared with the test which disregards the stratification, but it leads to no gain in power—the two tests become identical. Thus, the counter-intuitive suggestion that one should ignore the stratifying variable(s) would seem to be supported. However, as will be shown below, this result is model--dependent and is unique to the choice of the “canonical” logit link function.

### GENERAL LINK FUNCTIONS

A more general approach is to assume, in the population, a GLM, with binomial errors and arbitrary link function *g*():


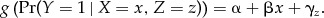


For example, the “liability threshold” model widely used in genetics can be represented in the above form by choosing the link function 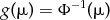, the inverse of the Gaussian probability distribution function. In the case-control study, we still have a GLM, but with a modified link function, 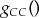, which now involves the ratio of sampling fractions, *K*:


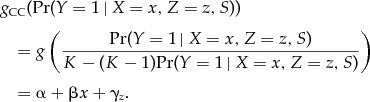


The first derivative of the log-likelihood function from the case-control study now becomes


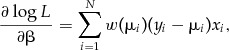


where, as before, 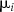 represents the fitted value of 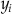 in the case-control study, and the weight function, *w*(), is given by


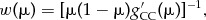


where 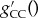 represents the first derivative of *g*_CC_(). In general, the weight function will depend on the ratio of sampling fractions, *K*.

When the logit link function applies in the population, the weight function is constant. Otherwise the efficient score tests will be weighted versions of those derived in the previous section. In particular, the statistic for the stratified test becomes


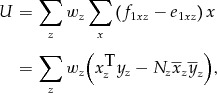


where the stratum weights are


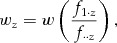


a function of the ratio of cases to controls in each stratum. When the stratifying variable is strongly related to disease, these weights could vary substantially when a link other than the logit is assumed. The variance of this statistic under the null (permutation) variance is


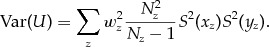


As before, when it can be assumed that *X* and *Z* are independent of one another, a more powerful test can be proposed. We simply replace the estimate of the conditional distribution of *X* given *Z* by the marginal sample distribution of *X*, and obtain


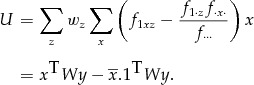


In the final expression, *W* represents a diagonal matrix with diagonal elements given by the appropriate 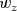. The appropriate null distribution for this test is generated by random permutations of the elements of *x* and 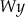. Its mean is zero and its variance is


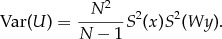


In effect, this test amounts to using the existing unstratified test, but with the 0/1 case-control indicator replaced by either zero (controls) or 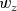 (cases). Thus, no new software will usually be required. A regression generalization follows naturally; instead of stratifying by a single discrete risk factor, we could regress phenotype on several known risk factors to obtain “fitted values,” μ, for each subject. The appropriate weight function 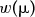 would then be used to generate subject-specific weights for later association tests.

### WEIGHTS FOR PROBIT AND OTHER LINK FUNCTIONS

The weight function derived in the last section is expressed as a function of the fitted value for the case-control indicator variable, μ, and the first derivative of the link function in the case-control study, 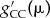. It can be convenient to rewrite this in terms of the risk in the equivalent stratum of the population, 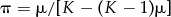, and the first derivative of the link function in the population model, 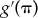:


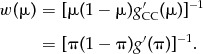


We shall denote this function by 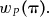


The weight functions corresponding to some important link functions are shown in Table 1.

**Table 1:** Stratum weights as a function of population risk

Link	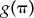	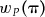
Logit	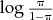	1
Log	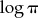	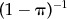
Probit	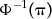	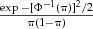
Identity	π	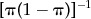
Independence	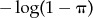	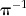
Power odds	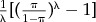	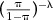

**Fig. 1:**
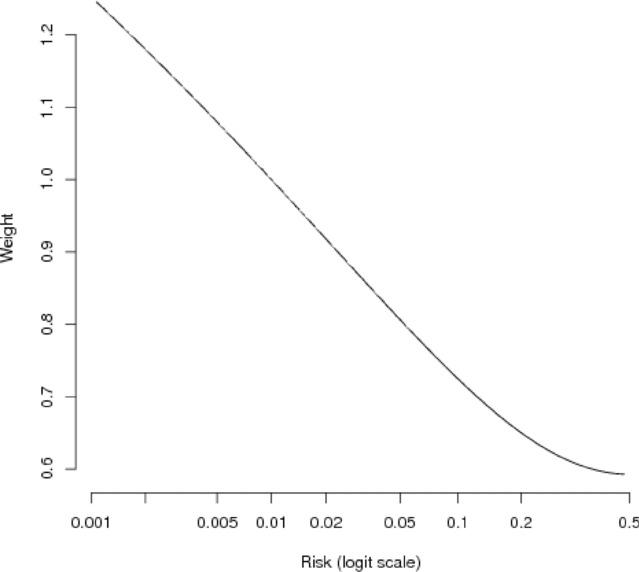
The probit link weight function for population risks in the range (0.001, 0.5), scaled relative to the weight for a risk of 0.01.

The probit link is of special interest. Its weight function for risks in the range (0.001, 0.5) is shown in Figure 1.

It is approximately linear in logit(π), and strata in which the population risk is low are given somewhat greater weight. Another link of special interest is the “independence” link function which, as we shall see, is of special interest as a model for the absence of epistasis. In that case, the weight function is simply 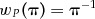 so that, again, greater weight is given to low-risk strata (although to a much greater extent than for the probit link). For small population risks (the position we are usually faced with), the independence link is closely approximated by the identity link and the log link is closely approximated by the logit link.

The “power odds” link function was discussed in the context of case-control studies by Breslow and Storer (1985). This general family of link functions leads to a wide range of weighting schemes:

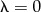
: (logistic model) the score test is unweighted;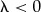: (risk accumulates less fast than the logistic model) low-risk strata are up-weighted in the test; and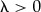: (risk accumulates faster than the logistic model) high-risk strata are up-weighted in the test.

This family of link functions is particularly convenient here in that 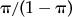 can be replaced by 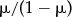, thus avoiding the need to estimate the sampling ratio. The stratum weights are simply a power of the stratum-specific case:control ratios. The probit link function is not far from linearly related to the logit link, but most closely linear with the power odds link with 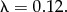


### POWER

This section explores the extent to which the weighting scheme is likely to be important in real studies. Two scenarios are considered:

1. (Large risk differentials). The population is assumed to fall into seven equally populated risk strata with risks 0.1, 0.05, 0.02, 0.01, 0.005, 0.002, and 0.001—a 100-fold variation.
2. (Moderate risk differentials). The population is assumed to fall into four equally populated risk strata with risks 0.05, 0.02, 0.01, and 0.005—a 10-fold variation.

In both cases, the non-centrality parameter of the Chi-squared test on 1 degree of freedom, 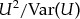, was calculated for a very small effect of a diallelic locus, with minor allele frequency 0.5, assumed constant across strata.

**Fig. 2:**
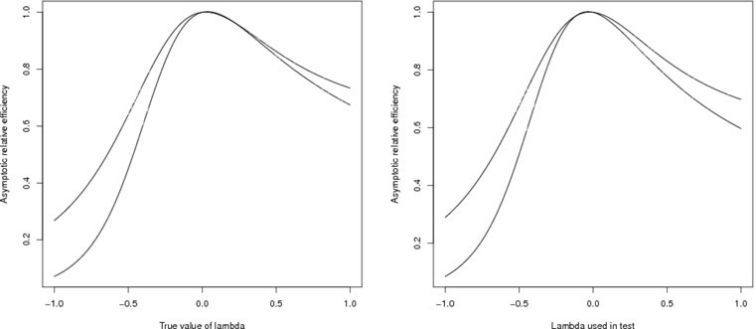
Efficiency loss due to misspecification of the link function (power odds family).

Figure 2 shows the effect of misspecifying the link function in the context of the power odds family. In the left-hand panel, datasets are generated using different values of λ and analysed with 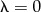 (which, as shown above, is equivalent to the standard Cochran-Armitage test). The efficiency measure plotted is the ratio of the non-centrality parameter for the Chi-squared (1 df) test to its value when the link is correctly specified. The lower curve refers to the more extreme scenario 1, and the upper to scenario 2. When the data are generated with 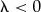, corresponding to accumulation of risk being *faster* than multiplicative, the standard Cochran-Armitage test can be quite inefficient. For positive values of λ, corresponding to submultiplicative risk accumulation (with 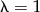 approximating additivity of risks), the loss of power is less extreme, although it can still be substantial. The right-hand panel of Figure 2 shows the same index when the data were generated according to the logistic regression model, but different values of λ are used to calculate weights.

The difference between logit and probit links is quite modest. The relative efficiency when using a Cochran-Armitage test when the data were generated by the probit (underlying liability) model is 0.949 in scenario 1 and 0.969 in scenario 2. The corresponding values when the data were generated by the logistic model and analysed with probit weights are 0.983 and 0.984.

**Fig. 3:**
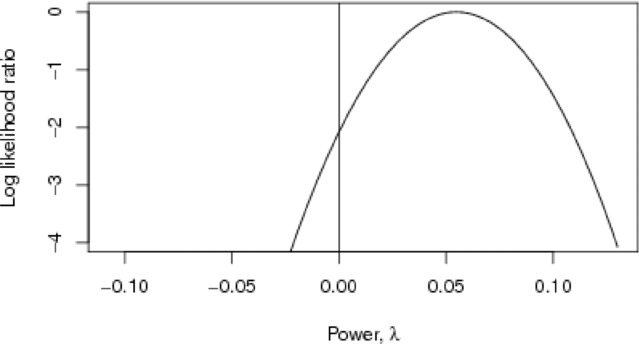
Log-likelihood for the parameter, ***λ***, of the power odds link for a case-control study of type 1 diabetes.

As indicated earlier, a possible application of these methods is the search for new disease susceptibility loci for type 1 diabetes. Here there is a very strong *HLA* association and some suggestion in the literature that additional effects tend to contribute in a submultiplicative manner. Figure 3 shows the results of fitting the power odds model in a large case-control study (Clayton, 2009).

For each value of λ, the regression model including all known disease susceptibility loci was fitted using standard generalized linear modelling software, and the figure shows the profile log-likelihood function for λ. The maximum-likelihood estimate was 0.05, corresponding to a (slightly) submultiplicative risk accumulation part-way between logit and probit links, although both of these links would be rejected at at the 0.05 level by a likelihood ratio Chi-squared test. The model of additive risk contributions (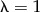) is clearly unsupported.

## INTERACTION AND EPISTASIS

The concept of link functions is also highly relevant to the continuing debate concerning the role of “epistasis” in complex disease genetics. In particular, it is widely believed that both failure to find new disease associations and poor prediction from known loci are attributable to a failure to allow for epistasis. The results of previous sections have considerable relevance to this suggestion.

It has frequently been pointed out that the genetics literature is confused on the topic of epistasis(see, e.g., Phillips (1998) and Cordell (2002)), but misconceptions due to a confused use of language still remain. The term was coined, by Bateson, in the context of fully penetrant traits whose inheritance could not be explained by action of a single locus. But, for traits which are not fully penetrant, the phenomenon becomes much more difficult to define precisely. In considering quantitative traits (which, by their nature, are not fully penetrant), Fisher (1918) introduced the term “epistacy” to refer to statistical interaction, or non-additivity, of the effects of two or more loci, in the same way as he used the term “dominance” to refer to non-additivity of the effects of the two alleles at a single autosomal locus. However, although there are clear analogies between the quantitative measures introduced by Fisher and the related concepts for fully penetrant traits, they are not the same thing and the resultant blurring of the language has not been helpful. Indeed, Fisher's epistacy has widely been referred to as epistasis, adding to the confusion. The problem of defining these concepts in terms of statistical interaction is that statistical interaction is scale-dependent; if two factors act additively on one scale of measurement they will interact, in the statistical sense, on a different scale. The same problem has been faced by epidemiologists in considering the question of the independence of causes in multifactorial disease and the issue has been widely debated in that literature (Thompson, 1991).

For incompletely penetrant dichotomous traits, Risch (1990) considered the role of epistasis both in determining the pattern of recurrence risks in extended families, and for the power of linkage studies. In these detailed studies, he defined absence of epistasis in terms of locus heterogeneity. This corresponds with the epidemiological concept of independent sufficient causes; if one factor acting alone causes a disease with probability γ_1_ and a second factor, also acting alone, causes the disease with probability γ_2_, then to remain free of disease one must avoid both causes and, if the causes are statistically independent, 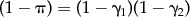. Writing 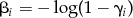, we then have


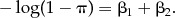


This is an additive model with link function 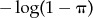 (referred to above as the “independence” link function). For small π, the usual position for disease traits, the independence link is closely approximated by the identity link. Thus, in the model of Risch, absence of epistasis corresponds closely to additivity of effects on risk itself.

In contrast, Risch chose to model epistatic action of two factors in terms of the multiplicative model for risks. A heuristic for this model is to consider the case in which the two causes considered in the previous paragraph must *both* occur for the disease to be penetrant. Then, again assuming independence of causes, 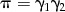 and, writing 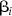 instead of 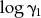,


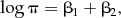


a model for additivity of effects on the log scale. Risch showed that relative recurrence risks fell much more sharply with distance of relationship to proband under this epistatic model than under the additive, non-epistatic model.

When the attention of scientists studying the genetics of complex disease switched to association studies, in particular to case-control studies, the obvious statistical approach was regression analysis and, for reasons given earlier, the most natural choice of regression model is logistic regression. Many authors, perhaps following Fisher, then started to use the term epistasis to refer to statistical interaction in the logistic regression model. Since, for small π the logit link is nearly the same as the log link, from this standpoint *absence* of epistasis corresponds to the model of multiplicative effects—precisely the model used by Risch as a model for epistatic action.

When, in some fields, the strategy of testing loci for association one-at-a-time led to disappointing results, the argument was frequently advanced that, since complex diseases clearly must involve interactions of causes in a mechanistic sense, association tests should allow for epistasis. Here, however, the results of previous sections are instructive; these show that the strategy of disregarding other loci when testing a new locus of interest is optimal when there is no interaction on the logit scale, and this corresponds with Risch's model for epistasis. Ironically, with the disease model of independent sufficient causes, Risch's model for absence of epistasis, the strategy of ignoring other loci is no longer optimal; subjects carrying high-risk alleles at other loci should be given less weight in the analysis. However, Figure 2 shows that very substantial loss of power due to the one-at-a-time strategy only occurs when effects are supra-multiplicative (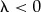), and it is not clear whether such interaction is widespread for complex traits.

In the context of the model of an underlying normally distributed liability, it is natural to regard interaction on the probit scale as a natural generalization of Fisher's “epistacy.” But there would seem to be no reason to believe that such interaction represents epistasis in any mechanistic sense. In the simple conceptualization of the model of no interaction on the probit scale, different causes contribute independently and additively to some underlying latent construct (liability), and disease follows when this exceeds a threshold. But this does not correspond to a model of independent sufficient causes; indeed, since the causes are acting in concert within a single mechanism, this model could be thought of as a model of epistasis rather than a model for no epistasis.

## PREDICTION

The question of the ability to predict, from their genotypes, those individuals who will develop various complex diseases has important implications for the future of health care, and has received some recent attention. Much depends on the manner in which risk accumulates due to the accumulation of multiple risk alleles. The link function provides a simplified way of addressing this problem, hopefully to guide intuition. The question of interest is, for a disease with a given heritability (loosely defined), just how predictable would disease occurrence be if we typed every disease susceptibility locus responsible? Here, the measure of heritability used will be the sibling relative recurrence risk, 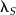, defined as the ratio of the risk to the sibling of an affected proband to the general population risk, and predictability will be the receiver operating characteristic (ROC) curve—the plot of the true positive probability (sensitivity) vs. the false positive probability for different thresholds for a genotype score.

### THE MULTIPLICATIVE (LOG LINK) MODEL

A multiplicative model for risks, appropriate for rare diseases, assumes that the multiple genes contribute to a genetic risk score, assumed to be approximately normally distributed in the population, risk being related to this genetic risk score via a log-link function. This model has been explored by Pharoah et al. (2002) and by Clayton (2009).

**Fig. 4:**
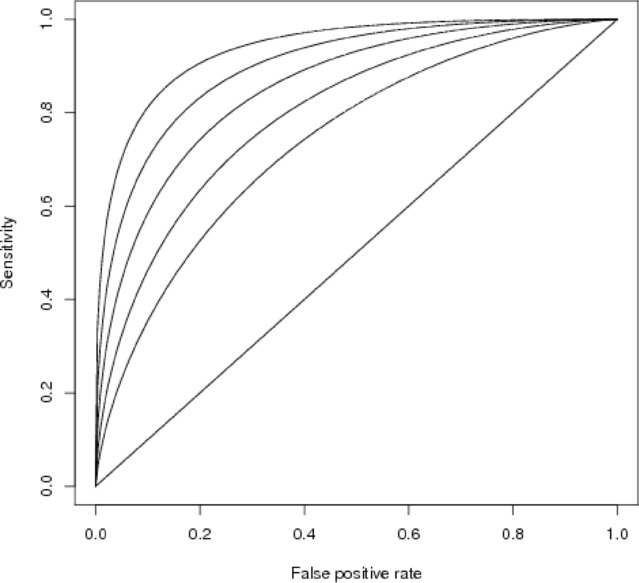
ROC curves for the log-link model for ***λ_S_*** of 1.5, 2, 3, 5, and 10. The population risk, ***P***, is taken as 0.005.

If the distribution of a genetic score, *x*, in the population is *N*(0,1), and the risk conditional on *x* is given by


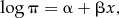


then the marginal population risk of disease is 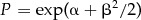 and the sibling recurrence risk ratio is 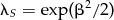. The distribution of the genetic risk score conditional upon disease occurrence is 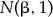, thus allowing plotting of ROC curves (Figure 4).

This model has been criticized by Wray et al. (2010), largely on the grounds that it can lead to predicted risks which exceed 1.0. However, this is unlikely to be a serious problem for most complex disease traits. For example, for 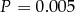 and 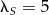, only a population fraction of 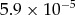 would have predicted risks greater than 1.0.

### THE INDEPENDENT SUFFICIENT CAUSES MODEL

A tractable independent sufficient causes model assumes

1. the number of risk variants, *x*, inherited by an individual follows a Poisson distribution with mean μ;
2. each variant, acting alone, would have penetrance *p*; and
3. there exists a background non-heritable (sporadic) risk, *b*, acting as a further independent sufficient cause.

Under the independent sufficient causes model, the causes act additively with link function 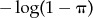:


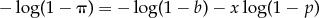


and, summing over the Poisson distribution of *x*, the marginal population risk becomes


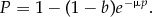


The numbers of risk alleles inherited by two siblings, *x*_1_ and *x*_2_, may be decomposed into a shared set and two residual unshared components, these three counts following Poisson distributions with means 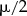. Under this model, the sibling recurrence risk ratio becomes


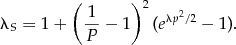


The ROC curves for this model may be simply described; for even moderately high values of 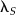 (>2, say), the curve steps to 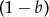 in a single step, thereafter following a straight line to the (1, 1) corner. In effect, the non-sporadic cases are predicted perfectly.

**Fig. 5:**
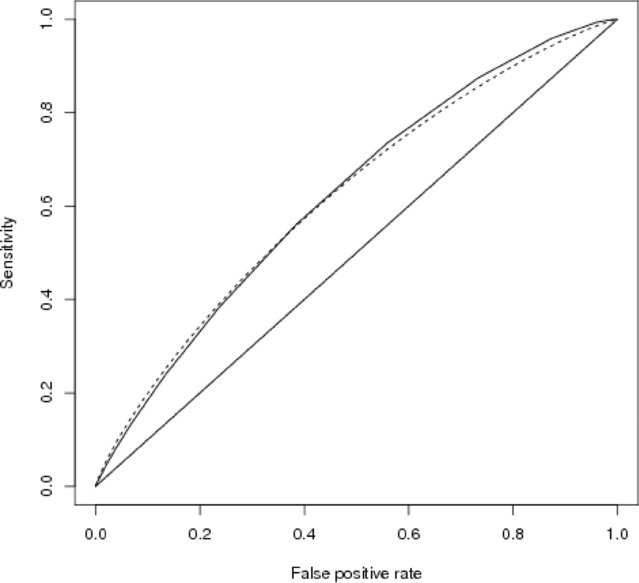
ROC curves for the independent sufficient causes link (solid line) and the log link (dashed line) for 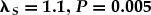.

This behaviour reflects the fact that the model, in effect, reverts to the single gene model with locus heterogeneity. To explain relatively high values of 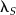, the penetrance of each causal genetic variant must be high and, to explain the relatively low population prevalence of disease, these variants must be rare. As a result, non-sporadic cases will have at least one variant (and rarely more than one), while non-cases will be very unlikely to carry any such variants. Prediction from genotype will be extremely accurate. However, when the value of 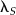 is little above one, this is no longer the case and prediction is little better than when a multiplicative model holds. For example, with 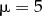, 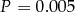 and no sporadic cases (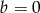), this model yields 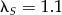. The mean number of causal variants carried by cases is ∼6 and, by non-cases, ∼5. The ROC curve is little different from that of the log-link model for the same value of 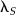 (Figure 5).

### THE POWER LINK

Further light is cast on the impact of the link function on the ability to predict a disease trait from genotype by considering the power transformation. For simplicity, consider a rare disease so that the power odds link can be replaced by the the power transformation of risk itself so that


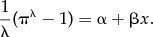


For small λ, this can be approximated by a quadratic model with log link:


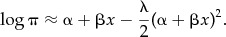


**Fig. 6:**
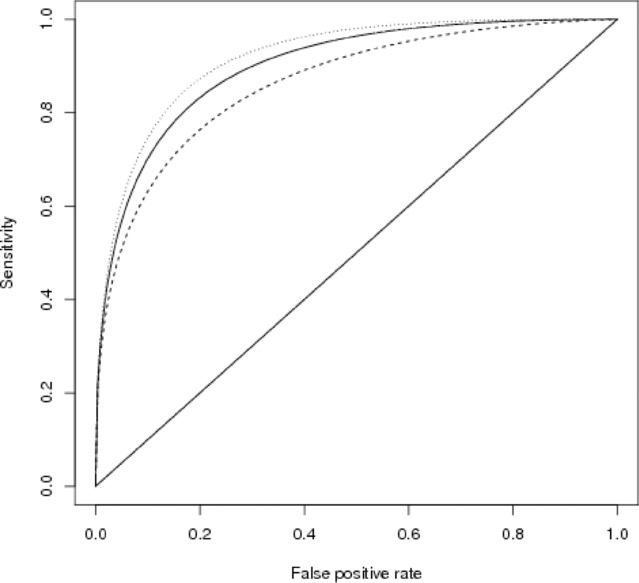
ROC curves for power link. Solid line has 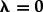 (multiplicative model), dashed line has 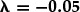, and dotted line has 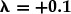 (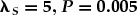).

When the population distribution of *x* is normal and risks are small, this yields tractable expressions for relative recurrence risks and for ROC curve calculations. Figure 6 shows ROC curves for the multiplicative model (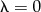) and for small positive and negative deviations from it.

For the submultiplicative model (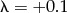), prediction is improved with respect to the multiplicative model while, for the supra-multiplicative model (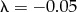), prediction is worse.

### THE PROBIT MODEL

The probit model also leads to tractable estimates for the ROC curve given availability of an algorithm to estimate the bivariate normal integral (Wray et al., 2010). If genotype and environment scores, *g* and *e* say, are distributed as *N*(0,1) and heritability of liability is *H* then liability, defined as 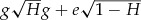, is also *N*(0,1). For marginal population risk *P*, the liability threshold is 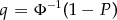 and, since the correlation between liabilities of two siblings is 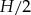, the sibling recurrence risk ratio is 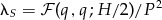, where


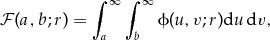


ϕ denoting the bivariate normal distribution function with unit variances and correlation *r*. Since genotype score, *g*, has correlation 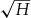 with liability, the cumulative distribution function of genotype score in cases is


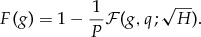


**Fig. 7:**
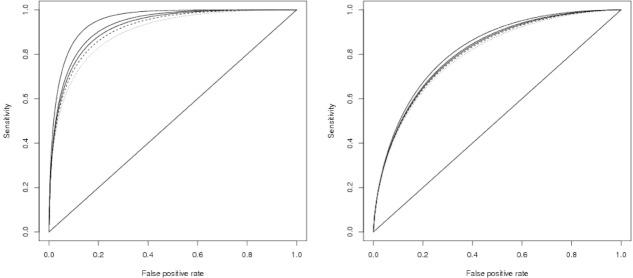
ROC curves for probit model for 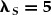 (left) and 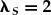 (right), and for ***P = 0.01, 0.001***, and 0.0001 (solid lines, top to bottom). The dotted line is for the multiplicative model with ***P = 0.0001***, and the dashed line is for the corresponding approximate power link model with power, 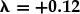.

Figure 7 compares some ROC curves for different values of 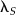 and *P*. For 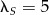, as was pointed out by Wray et al. (2010), prediction improves markedly with increasing population prevalence *P* and, even when *P* is very low, is significantly better than in the case where the multiplicative model holds. The approximate curve for the power link approximating to the probit (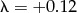) provides a fair approximation. When 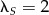, the five curves are much closer to one another.

The explanation for these results follows from the form of the probit link. We would expect to see a similar pattern for the logit link, another sigmoid function (although this case does not lead to tractable calculations). For small risks, these models are close to the multiplicative model for risks while, for larger risks the probit and logit links become nearly linear, approximating the additive model for risk. For risks close to one, the models are close to the independent sufficient causes model (i.e., multiplicative in 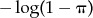). As we have seen, prediction is much better when an additive or independent sufficient causes model holds. Thus, prediction can be better under the probit model (and, one would presume, under a logistic model) because, for larger risks, it becomes closer to the additive risks model.

## DISCUSSION

We have shown that the strategy of ignoring other known disease susceptibility loci and risk factors when testing for new associations with complex disease, for example in genome-wide association studies, is justifiable, but only when effects combine additively on the logistic scale. More generally, weighted analyses may be appropriate, but this raises the question of how the weighting scheme might be chosen. This problem is ubiquitous in the choice of statistical tests: the optimal choice of test depends on the unknown state of nature. We have past experience to guide us so that, as in the case of type 1 diabetes (Figure 3), it is possible to estimate a link function by choosing from a general family such as the power-odds family using current data. Even here, however, the fact that one link function is generally most appropriate does not guarantee that it will always be optimal. Although, estimation of the link function from available data would probably be advocated as the best strategy, in the analysis of a genome-wide study there is a case for repeating the analysis with several plausible link functions. Although this could marginally increase the false positive rate such studies will, in any case, require replication.

It is commonly believed that underlying mechanisms for such diseases must involve “epistatic” action and, therefore, that statistical interactions must be widespread. However, the concept of “epistasis” cannot be simply identified with statistical interaction. Indeed, the logistic regression model with no statistical interaction between genes is quite strongly epistatic. The need to allow for other factors in carrying out association tests is particularly pressing in the presence of epistasis which is “supra-multiplicative,” in the sense that the joint effect of multiple factors exceeds the *product* of their effects when acting alone.

The precise scale on which multiple factors combine can also be important for assessing the potential for prediction of a trait from genotype, given its heritability (as assessed by recurrence risk ratios). Although the link function does not seem to be very important for a trait which is not very heritable, it can have quite an influence for a strongly heritable trait. If factors act supra-multiplicatively, phenotype is *less* predictable from genotype while, when joint effects are less than the product of single effects, phenotype is potentially more predictable from genotype. These considerations go some way to explaining the differing instincts of geneticists, who would expect a phenotype with high monozygotic twin concordance rate to be highly predictable, with those of epidemiologists familiar with the “prevention paradox” (Rose, 1992), who are more sceptical—in many multifactorial diseases, most cases derive from the bulk of the population at only modestly increased risk. It is too early to say which viewpoint will be appropriate for most common complex traits, although an epistatic model might be judged rather more plausible in most cases and this would favour the more sceptical view.
